# Draft Macronuclear Genome Sequence of the Ruminal Ciliate Entodinium caudatum

**DOI:** 10.1128/MRA.00826-18

**Published:** 2018-07-12

**Authors:** T. Park, S. Wijeratne, T. Meulia, J. Firkins, Z. Yu

**Affiliations:** a Department of Animal Sciences, The Ohio State University, Columbus, Ohio, USA; b Molecular and Cellular Imaging Center, Ohio Agricultural Research and Development Center, The Ohio State University, Wooster, Ohio, USA; c Department of Plant Pathology, The Ohio State University, Wooster, Ohio, USA; University of California, Riverside

## Abstract

The transcriptionally active macronucleus of a ruminal ciliate, Entodinium caudatum MZG-1, was sequenced using the Illumina MiSeq and Oxford Nanopore MinION platforms. This is the first draft macronuclear genome sequence of a ruminal protozoon, and the genomic information will provide useful insight into the metabolism, physiology, and ecology of ruminal ciliates.

## ANNOUNCEMENT

Entodinium caudatum, found only in the rumen or similar environments, is the most researched ruminal protozoal species because of its predominance (1) and high bacterivorous activity, which increases intraruminal nitrogen recycling and decreases nitrogen utilization efficiency (2–4). However, the inability to establish an axenic culture of any ruminal ciliate species has hindered understanding of their metabolism, physiology, and ecology and thus their actual roles in the rumen (5–8). Therefore, we sequenced the transcriptionally active macronuclear (MAC) genome of E. caudatum to help us understand its genomic and biological features.

E. caudatum strain MZG-1, initially isolated from the rumen of a gerenuk, was grown as a monoculture (in terms of ruminal protozoa) in SP medium (9). Cells of E. caudatum strain MZG-1 were separated from the associated prokaryotic cells present in the monoculture by sequential filtration and washing through four nylon filter membranes of decreasing pore size. Macronuclei were isolated following cell lysis (10, 11) and then purified using Percoll gradient centrifugation. Following confirmation by PCR amplification of the actin gene, MAC DNA was extracted using a QIAamp DNA minikit, and RNA was removed using DNase-free RNase. The MAC DNA was sequenced using the Illumina MiSeq and Oxford Nanopore MinION platforms. Approximately 40 million paired-end (2 × 300-bp) reads (∼6 Gb) were generated with the MiSeq platform, and 0.4 million 2D reads (∼0.78 Gb) were generated with the MinION platform (12). Trimmomatic version 3.2.2 was used to trim the Illumina adapters and filter out the reads with a Phred quality score of <20 for a 4-bp window (13). The quality-checked MiSeq reads (73,209,090 in total) were then assembled *de novo* using SPAdes (14). The quality-filtered MinION 2D reads (*Q* > 9) and the contigs assembled from the MiSeq reads were assembled again using SSPACE-LongRead version 2.0 (15). The final assembled draft MAC genome sequence (107,579,855 bp) had a coverage reaching 79× from a total of 20,400 contigs, with the *N*_50_ value being 9,873 bp and the longest contig being 147,117 bp. Putative telomeric repeats (5′-CCCCAAT)_n_ were searched from the E. caudatum MAC scaffolds using SCAMPI (16), and 1,307 scaffolds were capped with the putative repeats at both ends, while 5,911 scaffolds were capped at one end. Approximately 100 bp of subtelomeric regions with 15-bp AT periodicity was detected.

Genes were predicted *de novo* using AUGUSTUS (17) trained with the Tetrahymena thermophila MAC genome sequences, followed by annotation of the protein-coding sequences using Blast2GO and InterProScan version 5 (18–20). In total, 15,544 different protein-coding genes were annotated. The E. caudatum MAC genome sequence shared the most orthologs (698 in total) with that of Oxytricha trifallax, which has 16,000 chromosomes (21). Further analyses of the draft MAC genome and comparison with that of other ciliates will help us better understand the metabolism, physiology, and ecology of E. caudatum and its importance in rumen function and the interaction with other members of the rumen microbiome.

### Data availability.

This draft MAC genome of E. caudatum MZG-1 has been deposited in DDBJ/ENA/GenBank under the accession number NBJL00000000. The version described here is the second version, NBJL02000000.
